# Expert Involvement and Adherence to Medical Evidence in Medical Mobile Phone Apps: A Systematic Review

**DOI:** 10.2196/mhealth.4169

**Published:** 2015-07-27

**Authors:** Yousif Subhi, Sarah Hjartbro Bube, Signe Rolskov Bojsen, Ann Sofia Skou Thomsen, Lars Konge

**Affiliations:** ^1^ Centre for Clinical Education The Capital Region of Denmark Copenhagen Denmark; ^2^ Clinical Eye Research Unit Department of Ophthalmology Copenhagen University Hospital Roskilde Roskilde Denmark; ^3^ Department of Ophthalmology Copenhagen University Hospital Glostrup Glostrup Denmark

**Keywords:** mHealth, mobile apps, technology

## Abstract

**Background:**

Both clinicians and patients use medical mobile phone apps. Anyone can publish medical apps, which leads to contents with variable quality that may have a serious impact on human lives. We herein provide an overview of the prevalence of expert involvement in app development and whether or not app contents adhere to current medical evidence.

**Objective:**

To systematically review studies evaluating expert involvement or adherence of app content to medical evidence in medical mobile phone apps.

**Methods:**

We systematically searched 3 databases (PubMed, The Cochrane Library, and EMBASE), and included studies evaluating expert involvement or adherence of app content to medical evidence in medical mobile phone apps. Two authors performed data extraction independently. Qualitative analysis of the included studies was performed.

**Results:**

Based on inclusion criteria, 52 studies were included in this review. These studies assessed a total of 6520 apps. Studies dealt with a variety of medical specialties and topics. As much as 28 studies assessed expert involvement, which was found in 9-67% of the assessed apps. Thirty studies (including 6 studies that also assessed expert involvement) assessed adherence of app content to current medical evidence. Thirteen studies found that 10-87% of the assessed apps adhered fully to the compared evidence (published studies, recommendations, and guidelines). Seventeen studies found that none of the assessed apps (n=2237) adhered fully to the compared evidence.

**Conclusions:**

Most medical mobile phone apps lack expert involvement and do not adhere to relevant medical evidence.

## Introduction

### Background

Mobile health is growing [1]. Mobile apps are frequently used in daily clinical practice and enable immediate on-the-go access to key clinical information that supports clinical decision making [2-5]. Patients use apps for disease information, screening, self-treatment, and management [6-9]. One may rightly ask, “Who provides us our app content?” Currently, anyone can publish medical apps. Although some app stores check for fulfillment of a number of technical criteria (eg, whether the app crashes upon launch), no one validates the medical content and no expert approval or peer-review systems exist. Consequently, there are apps with variable quality: opioid-conversion apps suggest medication doses that may threaten patient safety [10], asthma self-treatment apps contain potentially life-threatening information [11], and very few apps on cardiopulmonary resuscitation are actually designed according to existing basic life-support guidelines [12].

### Objective

From the aforementioned discussion, it is obvious that we need an overview of the literature to understand the extent of this problem. In this paper, we review studies that evaluate quality of medical apps by evaluating expert involvement or adherence of app content to medical evidence. We relate our findings to current initiatives that seek to encounter this problem.

## Methods

### Eligibility Criteria

We followed the Preferred Reporting Items for Systematic Reviews and Meta-Analyses (PRISMA) guidelines for reporting systematic reviews [13]. We included studies evaluating expert involvement or adherence of app content to medical evidence in medical mobile phone apps. The following studies were considered eligible: (1) investigating medical mobile phone apps within a predefined topic using a search strategy, and (2) assessing expert involvement or adherence to relevant medical evidence. Given that the definition of an expert and acceptable credentials may vary widely, we did not restrict the inclusion of studies to our own definitions of these concepts. Similarly, the degree of adherence to relevant medical evidence was not defined in advance; instead, we noted the included studies’ own definitions and judgments. Language was restricted to only English. Case studies and reviews of a single app were excluded, because they did not include a search strategy to systematically review available apps.

### Search Strategy and Study Selection

We searched existing literature through the bibliographic databases PubMed, The Cochrane Library, and EMBASE using the following search terms: (“smartphone” OR “iPhone” OR “Android”) AND (“app” OR “application”). This broad search string was used to identify as many relevant studies as possible. The last search was performed March 17, 2015. One researcher (YS) removed all duplicates and screened all abstracts. All potentially eligible studies were read in full by 2 independent researchers (YS and SRB). Disagreements were resolved by discussion. References of all included studies were read to find additional eligible studies. We only included studies with original data.

### Data Collection and Synthesis of Results

The research group piloted a data-extraction form. We extracted information on topic, app stores searched, methods used for assessment of expert involvement/adherence to medical evidence, and study results. Two researchers (YS and SHB) extracted data independently. Disagreements were solved through discussion and consensus. Microsoft Excel (Redmond, WA, USA) was used for data collection and management. The heterogeneity of the studies did not permit pooling of study results to conduct a meta-analysis. All studies were included in a qualitative analysis.

## Results

### Studies Identified

**Figure 1:**
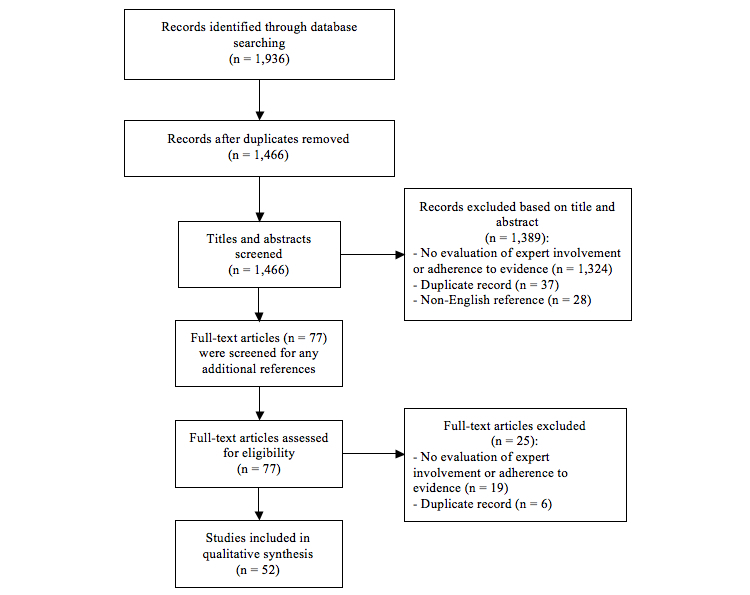
Preferred Reporting Items for Systematic Reviews and Meta-Analyses (PRISMA) flow diagram of search results and study selection.

The broad search strategy yielded 1936 records, of which a great number were duplicates or irrelevant (eg, mobile-phone-assisted data collection in biomedical research). Fifty-two studies were identified as relevant, and included in this review. These studies assessed 6520 apps. Details on search and study selection are presented in Figure 1.

Included studies are presented in Tables 1 and 2. Topics tended to be broader for studies of expert involvement (eg, dermatology [14], ophthalmology [15], or pain management [16-18]), whereas studies on adherence to medical evidence tended to be more specific (eg, asthma self-management [11], prostate cancer [19], pediatric obesity [20,21]). Studies included a median of 71 apps (interquartile range 41-148), and studies of expert involvement tended to have slightly higher number of included apps with a median of 85 apps (interquartile range 39-192 apps), compared with studies of adherence to medical evidence having a median of 63 apps (interquartile range 40-104 apps). Studies reviewed mostly included apps from the Apple App Store (n=49, 98%) and Google Android Market (n=36, 71%). Fewer studies included apps from less popular app stores such as BlackBerry Market (n=19, 38%), Windows Market (n=16, 32%), Nokia Ovi (n=11, 22%), and Samsung Market (n=9, 18%). Studies that included a search in these less popular app stores were often unable to find any relevant apps for inclusion [10,16,22-26].

### Studies on Expert Involvement

Twenty-eight studies assessed 3852 apps for expert involvement (Table 1). These studies dealt with topics within a variety of medical specialties and topics. The following 2 topics were assessed more than once: pain management (n=3) [16-18] and bariatric surgery (n=2) [22,23]. Studies mostly used the app stores’ app description (n=28, 100%) and the developers’ website (n=15, 54%) to determine whether an app had expert involvement. Nine studies (32%) also downloaded the apps. All studies found that at least some of the assessed apps had expert involvement and none found expert involvement in all assessed apps. Overall, expert involvement was found in 9-67% of assessed apps.

**Table 1:** Included studies with assessment of expert involvement.^a,b,c,d^

Reference	Topic	App stores in study^e^						Assessment based on			Apps in study	Expert involvement
		Apple	Google Play	BlackBerry World	Windows Phone Store	Nokia Ovi	Samsung	D^f^	W^g^	C^h^	N	n (%)
[27]	Addiction recovery	-	+/+	-	-	-	-	+	+	-	87	11 (12.6)
[22]	Bariatric surgery	+/+	+/+	+/-	+/-	-	+/-	+	+	-	83	32 (38.6)
[23]	Bariatric surgery	+/+	+/+	+/-	-	-	-	+	-	+	28	12 (42.9)
[28]	Breast diseases	+/+	+/+	+/+	+/+	-	-	+	+	-	148	19 (12.8)
[29]	Cardiothoracic surgery	+/+	+/+	-	-	-	-	+	+	-	379	78 (20.6)
[30]	Colorectal diseases	+/+	+/+	+/+	+/+	+/+	+/-	+	-	-	63	21 (33.3)
[31]	Contraceptive reminder	+/+	+/+	-	-	-	-	+	-	+	32	3 (9.4)
[32]	Depression	+/+	+/+	+/+	+/+	+/+	-	+	-	-	243	30 (12.3)
[14]	Dermatology	?	?	?	?	?	?	+	+	-	61	39 (63.9)
[33]	Headache	+/+	+/+	-	-	-	-	+	-	+	38	7 (18.4)
[34]	Hepatitis	+/+	+/+	-	-	-	-	+	-	+	23	13 (57.5)
[35]	Hernia	+/?	+/?	+/?	+/?	+/?	+/?	+	+	-	26	9 (34.6)
[36]	Human immunodeficiency virus/acquired immune deficiency syndrome	+/+	+/+	-	-	-	-	+	-	+	41	20 (48.8)
[37]	Medical hypnosis	+/+	-	-	-	-	-	+	+	-	407	141 (34.6)
[38]	Melanoma detection	+/+	+/+	-	-	-	-	+	-	-	39	4 (10.3)
[24]	Microbiology	+/+	+/+	+/+	+/+	+/-	+/-	+	+	-	94	32 (34.0)
[39]	Neurosurgery	+/+	+/+	-	-	-	-	+	+	-	111	73 (65.8)
[15]	Ophthalmology	+/+	-	-	-	-	-	+	-	-	182	68 (37.4)
[10]	Opioid conversion	+/+	+/+	+/+	+/+	+/-	+/-	+	+	+	23	11 (47.8)
[16]	Pain management	+/+	+/+	+/+	+/-	+/-	-	+	-	-	104	15 (14.4)
[17]	Pain management	+/+	+/+	-	-	-	-	+	+	+	12	2 (16.7)
[18]	Pain management	+/+	+/+	+/+	-	-	-	+	+	+	220	77 (35.0)
[40]	Pharmacology and drug prescription	+/+	+/+	+/+	+/+	+/+	+/+	+	+	-	306	206 (67.3)
[25]	Radiology	+/+	+/+	+/+	+/+	+/+	+/-	+	+	-	321	185 (57.6)
[41]	Stroke	+/+	+/+	-	-	-	-	+	-	-	93	44 (47.3)
[42]	Surgery	+/+	+/+	-	-	-	-	+	-	-	597	72 (12.1)
[43]	Urolithiasis	+/+	+/+	+/+	+/+	-	-	+	-	+	42	15 (35.7)
[26]	Vascular diseases	+/+	+/+	+/-	+/-	+/-	+/-	+	+	-	49	13 (26.5)

^a^ *+/+* indicates that the app store was searched and that apps were found.

^b^ *+/-* indicates that the app store was searched, but no apps were found.

^c^ *-* indicates that the app store was not searched.

^d^ ? indicates that whether or not the app store was searched was unclear.

^e^ We have included both the app stores searched and the app stores in which included apps were found.

^f^ App description from the app store

^g^ Developer’s website

^h^ Downloaded app content

### Studies on Adherence to Medical Evidence

Thirty studies assessed 3051 apps for adherence to medical evidence (Table 2). Six topics were investigated in more than 1 study: weight loss (n=4) [44-47], smoking cessation (n=3) [48-50], disease self-management (n=3) [11,51,52], pediatric obesity (n=2) [20,21], physical activity (n=2) [53,54], and sports injury (n=2) [55,56]. Remaining studies investigated apps on a diverse range of topics. Assessment was mostly based on downloaded app content (n=24, 86%). In 2 studies, it was unclear whether the assessment was based on downloaded app content [52,57]. Three studies only used the app stores’ app description for the assessment [38,44,58]. Studies compared the apps with a variety of forms of medical evidence. For example, smoking cessation apps were compared with US Public Health Service’s clinical practice guidelines for treating tobacco use and dependence [48,49]. Several studies correlated the app contents with available Cochrane reviews, other systematic reviews, or other published evidence [10,11,28,38,41,55,58-60]. In 6 studies, the assessment relied on criteria for ideal app contents as defined by the authors [33,61] or whether the app contents adhered to the general knowledge of the authors [19,43,46,62]. In 17 studies, none of the assessed apps (n=2237) adhered fully to the compared evidence [11,20,21,33,38,44-49,51-54,58,61]. In the remaining 13 studies, 10-87% of the assessed apps showed complete adherence to medical evidence [10,12,19,28,41,43,50,55-57,59,60,62]. Of these, only 5 studies found that more than half of the assessed apps showed complete adherence to medical evidence [19,41,56,60,62]; of note, 2 of these were based on the authors’ own self-stated expertise [19,62]. In most studies, a number of apps adhered partly to the assessed evidence. No topic was clearly associated with a higher or lower prevalence of adherence to available evidence—lack of adherence was highly prevalent in all studied topics.

**Table 2:** Included studies with assessment of adherence to available evidence.^a-d^

Reference	Topic	App stores in study^e^						Assessment based on			Adherence to evidence based on	Apps in study	Complete adherence^f^
		Apple	Google Play	BlackBerry World	Windows Phone Store	Nokia Ovi	Samsung	D^g^	W^h^	C^i^		N	n (%)
[58]	Alcohol use	+/+	-	-	-	-	-	+	-	-	Evidence-based principles from published reviews, from the website of the National Institute on Alcohol Abuse and Alcoholism and the American Psychological Association	767	0 (0.0)
[11]	Asthma self-management	+/+	+/+	+/+	+/+	-	-	-	-	+	Correlation with international guidelines, systematic reviews, and best practices	103	0 (0.0)
[28]	Breast diseases	+/+	+/+	+/+	+/+	-	-	+	+	-	Correlation with international guidelines, systematic reviews, and best practices	148	21 (14.2)
[62]	Cancer	+/+	-	-	-	-	-	-	-	+	The authors’ general knowledge on the area	77	42 (54.5)
[51]	Diabetes self-management	+/+	-	-	-	-	-	+	-	+	Inclusion of behaviors recommended by the American Association of Diabetes Educators	227	0 (0.0)
[59]	Eating disorders	+/+	+/+	+/+	+/+	+/+	-	+	-	+	Correlation with international guidelines, systematic reviews, and best practices	13	2 (15.4)
[33]	Headache	+/+	+/+	-	-	-	-	+	-	+	Criteria for an ideal app as defined by the authors	38	0 (0.0)
[57]	Hyper-tension	+/+	-	-	-	-	-	-	-	?	Conformity to guidelines	96	15 (15.6)
[52]	Hypertension self-management	+/+	-	-	-	-	-	+	-	?	Adherence to the Canadian Hypertension recommendations	58	0 (0.0)
[61]	Medication adherence	+/+	+/+	+/+	-	-	-	+	-	+	Ranking by authors’ consensus on desirable app content	147	0 (0.0)
[38]	Melanoma detection	+/+	+/+	-	-	-	-	+	-	-	Correlation with international guidelines, systematic reviews, and best practices	39	0 (0.0)
[60]	Oncology	+/+	+/+	+/+	+/+	-	-	-	-	+	Correlation with international guidelines, systematic reviews, and best practices	50	33 (66.0)
[10]	Opioid conversion	+/+	+/+	+/+	+/+	+/-	+/-	+	+	+	Assessment of whether the apps refer to any publication or source to the algorithms used	23	11 (47.8)
[20]	Pediatric obesity	+/+	-	-	-	-	-	+	-	+	Inclusion of recommended strategies and behavioral targets of the Expert Committee for Pediatric Obesity Prevention	57	0 (0.0)
[21]	Pediatric obesity	+/+	-	-	-	-	-	+	-	+	Adherence to American Academy of Pediatrics’ guidelines for the prevention of pediatric obesity	62	0 (0.0)
[53]	Physical activity	+/+	-	-	-	-	-	-	-	+	Rating on a scale from 0 to 100 based on inclusion of instruments from evidence-based behavior change theories	127	0 (0.0)
[54]	Physical activity	+/+	+/+	-	-	-	-	+	-	+	Rating based on the taxonomy of behavior change techniques used in interventions	64	0 (0.0)
[19]	Prostate cancer	+/+	-	-	-	-	-	-	-	+	The authors’ general knowledge on the area	15	13 (86.7)
[12]	Resuscitation	+/+	+/+	-	-	-	-	-	-	+	Adherence to the resuscitation guidelines from the European Resuscitation Council and American Heart Association	46	16 (34.8)
[48]	Smoking cessation	+/+	-	-	-	-	-	+	-	+	Coding according to items in the US Public Health Service's clinical practice guidelines for treating tobacco use and dependence	47	0 (0.0)
[49]	Smoking cessation	+/+	+/+	-	-	-	-	-	-	+	Coding according to items in the US Public Health Service's clinical practice guidelines for treating tobacco use and dependence	98	0 (0.0)
[50]	Smoking cessation	+/+	+/+	-	-	-	-	+	-	+	Inclusion of features that are in accordance with the self-determination theory	175	18 (10.3)
[55]	Sports injury	+/+	-	-	-	-	-	+	-	+	Correlation with available Cochrane reviews, other systematic reviews, or otherwise available evidence	18	5 (27.8)
[56]	Sports injury	+/+	+/+	+/+	+/+	+/+	+/+	+	-	+	Adherence to items in the Sports Concussion Assessment Tool 2	18	12 (66.7)
[41]	Stroke	+/+	+/+	-	-	-	-	+	-	+	Correlation with international guidelines, systematic reviews, and best practices	93	55 (59.1)
[43]	Urolithiasis	+/+	+/+	+/+	+/+	-	-	+	-	+	The authors’ general knowledge on the area	42	6 (14.3)
[44]	Weight loss	+/+	-	-	-	-	-	+	-	-	13 evidence-informed practices as defined by the Centers for Disease Control and Prevention, National Institutes of Health, the Food and Drug Administration, and the US Department of Agriculture	204	0 (0.0)
[45]	Weight loss	+/+	+/+	-	-	-	-	-	-	+	Inclusion of behavioral strategies from evidence-based weight loss interventions	30	0 (0.0)
[46]	Weight loss	?	+/+	?	?	?	?	-	-	+	The authors’ general knowledge on the area	65	0 (0.0)
[47]	Weight loss	+/+	-	-	-	-	-	+	-	+	Evaluation of the quality of health information using the Silberg scale	104	0 (0.0)

^a^ *+/+* indicates that the app store was searched and that apps were found.

^b^ *+/-* indicates that the app store was searched, but no apps were found.

^c^ *-* indicates that the app store was not searched.

^d^ Studies in which this is unclear is noted with “?”

^e^ We have included both the app stores searched and the app stores in which included apps were found.

^f^ Complete adherence is present for an assessed app when it meets the individual study’s definition of complete adherence to the relevant guidelines, recommendations or scientific content. As such, a “0” in this column means that no single app assessed met the criteria for complete adherence.

^g^ App description from the app store

^h^ Developer’s website

^i^ Downloaded app content

## Discussion

### Principal Findings

Medical apps may save lives; with no regulation of the content, however, we fear that they may also do harm. Studies in this review focused on a wide range of medical topics, app platforms, and assessment methods and all reached one general conclusion: medical mobile phone apps generally lack expert involvement and do not adhere to relevant medical evidence. Expert involvement was found in 9-67% of assessed apps. Adherence to medical evidence was found in 10-87% of the assessed apps in 13 studies, and in none of the assessed apps in 17 studies. Medical professionals and patients should be aware of this, as mobile phones increasingly play a role in medical education [5], clinical decision making [2], and patient empowerment [6-9].

For the common user, it may be practically impossible to assess whether or not an app adheres to current evidence and guidelines. In some cases, the app descriptions include references to publications from which the content is based. Levels of evidence as defined by the Oxford Centre for Evidence-Based Medicine state that systematic reviews and individual studies rank higher than opinions of an expert, but an expert opinion ranks better than nothing [63]. Hence, although expert involvement does not guarantee adherence to relevant medical evidence, it may be safer to have an expert involved than none.

Cheap and technically simple methods enable experts and clinicians to develop medical apps on their own [64-67]. These methods are based on Web apps developed using tools with a simple interface, hosted online, and distributed by the experts and clinicians [64-67]. Published examples include 1 Web app with clinical instructional videos for joint examination and 1 Web app with videos on psychiatric assessments and psychopathology lessons [65,67]. These works demonstrate that it is possible for experts to develop Web apps on their own with useful results [64,66]. However, 1 study in our review assessed both expert involvement and adherence of content to published evidence among opioid-conversion apps, and found that expert involvement per se does not necessarily lead to medical correctness of the content [10].

Apps can be considered an interactive way of communicating knowledge. We already use peer-review systems for such purposes—at least in scholarly journals—and one way of ensuring medically correct apps could be through peer reviews, which due to the unregulated nature of app stores would arrive after app publication. There are examples of short publications in medical journals of a review of 1 or more apps [68,69], and app developers are able to get an independent app review by submitting a request to Journal of Medical Internet Research mHealth and uHealth [70]. In addition, dedicated Web pages for app reviews exist [71,72]. One example of this is the Health Apps Library, which is developed and supported by the National Health Service in the United Kingdom [72]. The Health Apps Library enables developers to submit their app for review by clinicians that assesses whether the app is relevant to people in the United Kingdom, provide information from trusted sources, and comply with relevant data protection regulations [72]. The clinician then decides whether the app can be approved and published on the Health Apps Library [72]. However, even if a review exists, the user may not be aware of this. If the review is undesirable, the app developer may omit from referring to the review, which creates a bias. Previous studies on health information on the Internet reported similar results—some sources provide medically correct information, and some do not [73]—therefore, the problem highlighted in our systematic review is not new. However, some differences do exist when dealing with apps, which may allow to address this problem in the future. Apps are already reviewed by app stores before publication and app stores provide a streamlined access to content. Therefore, one possible way of addressing this problem could involve the collaboration between app stores and a regulatory third party such as the Health Apps Library when publishing apps with medical content.

### Limitations

Limitations of our approach should be noted. Apps can have expert involvement without stating it to the user, and app content may be accurate without referring to medical publications. In addition, apps with expert involvement can also contain inaccurate information, and referring to medical publications does not prevent out-of-date or inaccurate content. None of the studies included assessment of the actual use of the apps, which would provide an interesting dimension to our research question, as owning an app does not necessarily mean that the apps is used. These dimensions may be enlightened by future studies. Our review found that different methods were used for the assessment of expert involvement and medical adherence. Some studies assessed expert involvement or adherence to medical evidence only by reviewing the app descriptions in the app stores and by visiting the app developers’ website (Tables 1 and 2). For example, 1 study reviewed apps dealing with alcohol abuse and categorized each app’s approach using the app description [58]. We acknowledge that in some cases, this approach may provide sufficient results. However, one should note that app descriptions do not necessarily reflect the actual app content. Therefore, future studies are encouraged to download and review actual app content. A clear consensus on a methodological golden standard does not exist, but we are currently seeing inspiring studies that explore different methods that evaluate authorship and content [47,74]. One recent example is the Mobile App Rating Scale (MARS), a 23-item assessment tool that provides quality scores for an app within 5 dimensions (engagement, functionality, aesthetics, information quality, and subjective quality), which demonstrated a high level of internal consistency and inter-rater reliability [74]. Reliable tools such as the MARS are important for the future direction of how and what to review, and may help future research in providing more comparable results.

In conclusion, most medical mobile phone apps lack expert involvement and do not adhere to relevant medical evidence. Because mobile phones are highly prevalent among medical professionals and patients, this poses a significant problem. Review services do exist, but additional effort is needed, and attention to the problem may help the community to figure out the solutions of the future.

## Abbreviations

MARS: Mobile App Rating Scale
